# Clinico-epidemiological and treatment factors impact on survival in Egyptian patients with head and neck sarcoma: a retrospective case-series analysis

**DOI:** 10.1186/s43046-024-00242-2

**Published:** 2024-11-25

**Authors:** Mohamed Reda Kelany, Asmaa A. Abd Eltawab, Mohamed Naguib Mohamed, Mohamed Fathy Bayomy, Doaa Atef Mohamed Soliman

**Affiliations:** 1 Clinical Oncology, Faculty of Medicine Ain Shams University, Cairo, Egypt; 2 Clinical Oncology, Faculty of Medicine, Assuit University, Assuit, Egypt; 3 Otolaryngology-Head and Neck Surgery, Faculty of Medicine Ain Shams University, Cairo, Egypt; 4 Clinical Oncology, Faculty of Medicine, Zagazig University, EL-Sharqea, Egypt

**Keywords:** Head and neck sarcomas, Overall survival, Time to relapse

## Abstract

**Background:**

Head and neck sarcomas are very rare accounting for about 1% of head and neck malignancies and 5% of sarcomas. Outcomes have historically been worse in this group compared to other sarcomas, due to anatomical constraints that make complete surgical removal difficult and increased local relapse rate. Surgery remains the main intervention although the data suggest the role of chemotherapy and irradiation as treatment options.

**Methodology:**

and Design.

Data of patients diagnosed with head and neck sarcoma were retrospectively collected. Clinicopathologic characteristics and patients’ management were reviewed. Time to relapse (TTR) includes both time to local relapse (TTLR) and time to systemic relapse (TTSR). Overall survival (OS), TTLR and TTSR were calculated with Kaplan Mayer analysis using log rank test.

**Results:**

Twenty-four patients were retrospectively identified. Mean age was 37.7 years (range 17–80) with female gender predominance (62.5%). Rhabdomyosarcoma and osteosarcoma were the most common types (16.7%). At presentation, 23 patients showed localized disease (95.8%), while one patient was metastatic to the bone. Surgery was the primary treatment in 83.3% patients with different surgical margin status (R0 in 6/20, R1 in 11/20 and R2 in 3/20 patients), while 4/24 patients were not operated. Radiotherapy was applied as adjuvant treatment in 9 patients, as definitive in 2 and as palliative in one patient. Chemotherapy was administered in neoadjuvant/ adjuvant settings in 8 patients. Median follow-up was 31 months. Mean TTR for all surgically resected population was 39.8 months. Locally relapsed patients were 35% with mean TTLR 43.2 months while 15% of patients developed systemic relapse with mean TTSR 55 months. Mean OS of all studied patients was 48 months.

**Conclusion:**

Head and neck sarcomas are rare challenging malignancies due to anatomical constrain, with only 20% achieving R0 surgical resection and > 30% suffering of local relapse after complete surgical resection.

## Introduction

Sarcomas are divided into two types: soft tissue sarcomas and bone/cartilage sarcomas. They originate from mesenchymal cells and are a diverse group that arises from many different tissues [1]. Approximately 80% of sarcomas originate from soft tissues, while 20% arise from bone. Lesser than 5000 cases of sarcomas occur in the United States annually [2].

Sarcoma of the head and neck is rare accounting for 4–10% of all sarcomas [3]. Although more than 50 histological subtypes have been identified, the current staging criteria used to determine treatment are for almost all subtypes and depend on the histological grade, tumor size and depth, as well as the presence of regional lymphatic affection or distant metastasis. Also, they are grouped together because of similarities in prognostic factors, clinical presentation, and overall outcome [4].

Genetic diseases such as Li-Fraumeni syndrome or type-1 neurofibromatosis may induce certain sarcomas; and genomic abnormalities are frequently reported on molecular analysis [5]. Irradiation is associated with elevated incidence [6].

The general concepts of sarcoma management are not universally applied in head and neck. So, management presents a great challenge. The optimal treatment is complete surgical resection. The delicate anatomy of the head and neck limits the ability to obtain wide surgical margins. This may be the reason for high local recurrence rate and worse disease-specific survival compared to other sites [7].

Due to the rarity of head and neck sarcomas in adult, there is not enough clinical evidence-based data in the literature to provide sufficient patient’s number to identify prognostic factors influence on overall survival [4].

## Methodology and design

Data of patients diagnosed with head and neck sarcoma confirmed by histopathology from January 2015 to December 2021 were retrospectively collected. Clinicopathologic characteristics and patients’ management were reviewed. Time to relapse (TTR) includes both time to local relapse (TTLR) and time to systemic relapse (TTSR). Overall survival (OS), TTLR and TTSR were calculated from time of pathological diagnosis with Kaplan Mayer analysis using log rank test.

## Results

**Fig. 1:**
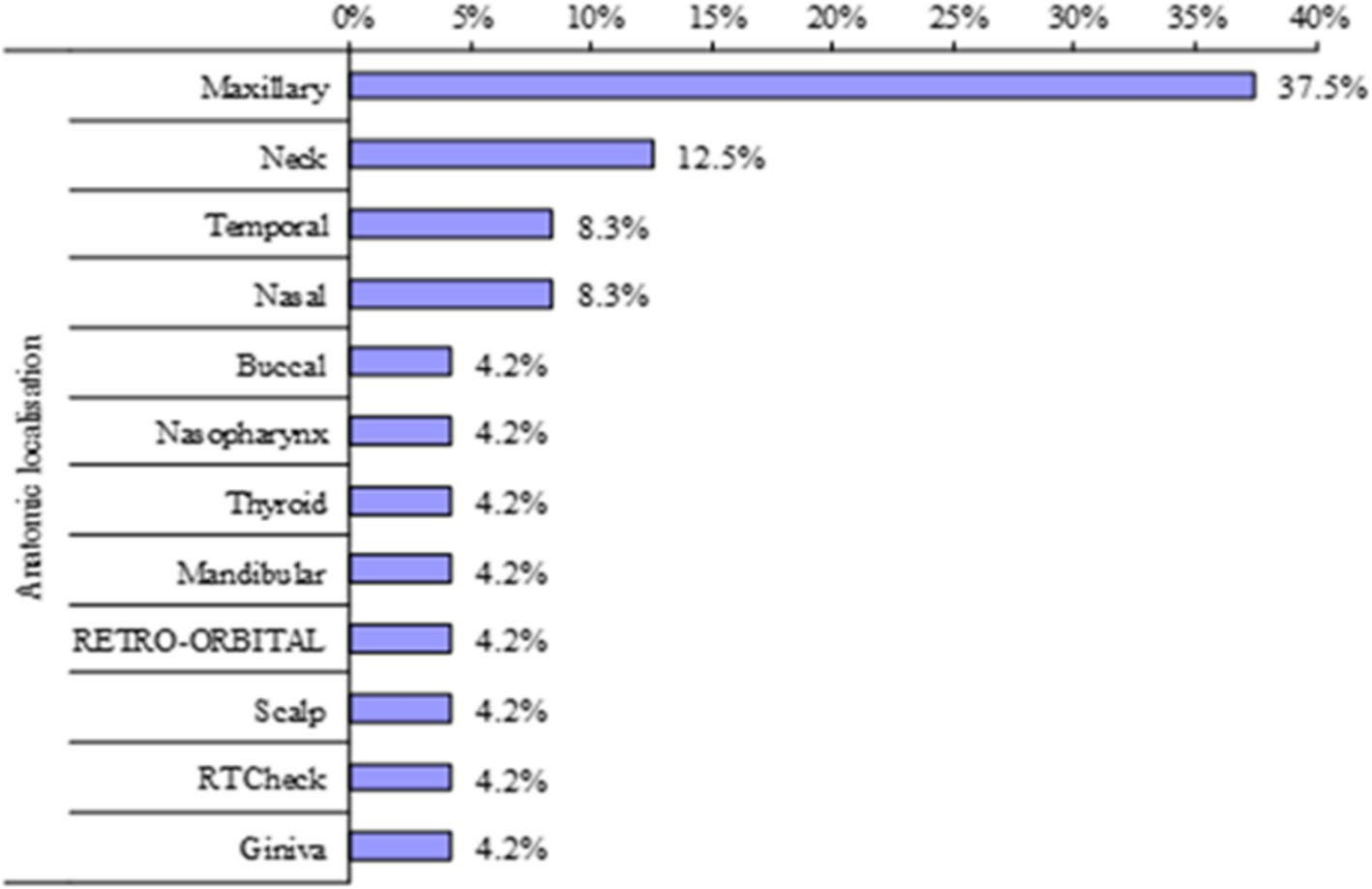
Shows anatomical sites of tumor presentation

Twenty-four patients were enrolled with mean age 37.7 years (range 17–80). Female gender was predominant (15/24) representing 62.5% (Table 1). The commonest site was maxilla as 9 patients presented with maxillary lesion (37.5%) divided as 3 maxillary osteosarcoma and 6 maxillary soft tissue sarcomas. Neck was the second common presented site (12.5%) (Fig. 1).

**Table 1:** Analytical data related to patients’ personal history, tumor site, diagnosis, and pathological type

		**No. = 24**	**%**
**Sex**	Female	15	62.5%
	Male	9	37.5%
**Anatomic localization**	Maxillary	9	37.5%
	Neck	3	12.5%
	Temporal	2	8.3%
	Nasal	2	8.3%
	Buccal	1	4.2%
	Nasopharynx	1	4.2%
	Thyroid	1	4.2%
	Mandibular	1	4.2%
	Retro-orbital	1	4.2%
	Scalp	1	4.2%
	RT Check	1	4.2%
	Gingiva	1	4.2%
**Biopsy**	No	8	33.3%
	Yes	16	66.7%
**Histologic diagnosis**	Rhabdymyosarcoma	4	16.7%
	Synovial sarcoma	2	8.3%
	Osteosarcoma	4	16.7%
	Angiosarcoma	2	8.3%
	DFS	2	8.3%
	Fibrosarcoma	2	8.3%
	Ewing	1	4.2%
	Chondrosarcoma	1	4.2%
	Fibromyxoidsarcoma	1	4.2%
	Epitheliod P. nerve sheeth sarcoma	1	4.2%
	Schwanoma	1	4.2%
	MYO6	1	4.2%
	Leiomyosarcoma	1	4.2%
	Myxo-inflammatory FIB	1	4.2%

**Fig. 2:**
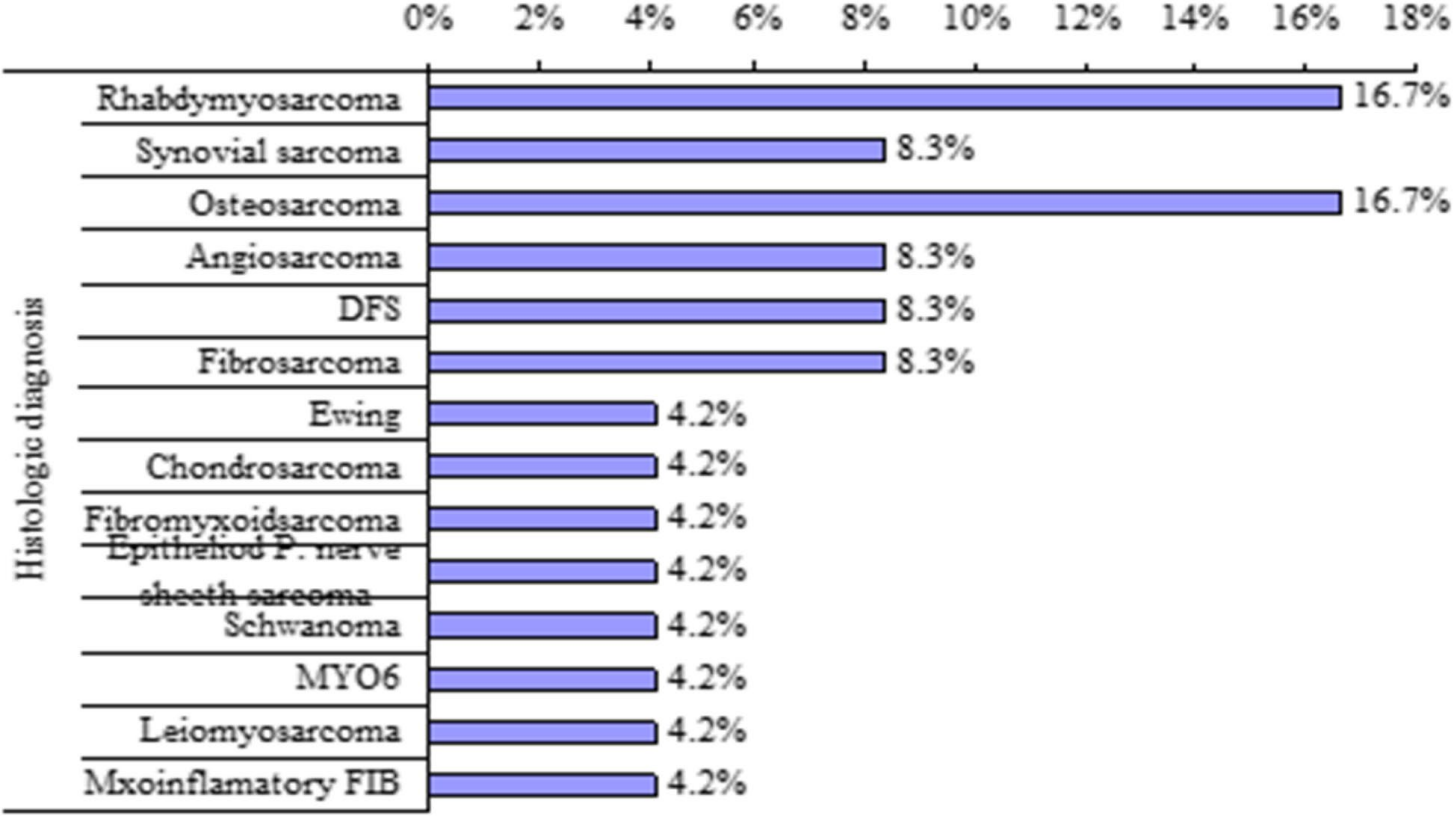
Shows pathological subtypes presentation

Rhabdomyosarcoma and osteosarcoma were the most common pathological subtypes with equal presentation by 4 patients for each (16.7%) (Fig. 2). Less common presentation of Dermatofibrosarcoma protuberans (DFS), angiosarcoma, synovial sarcoma and fibrosarcoma (8,3% for each).

Regarding TNM staging, T2N0M0 was most common presenting stage (13 patients). T2-stage was the tumor stage in 54.2% of patients and less common T3- stage in 25% of patients. Twenty patients presented with negative LN affection 20/24 (83.3%). At presentation, 23/24 patients showed localized disease (95.8%), while 1/24 patient (4.2%) was metastatic to bone. Mitotic count score 1 was more common than score 2 (58.3% vs 41.7%). Fourteen patients’ tumor samples showed no tissue necrosis by pathological examination. Grade II had the upper hand by both TNM grading and FNCLCC grading (66.7%, 45.8% respectively) (Table 2).

**Table 2:** Analytical data related to tumor stage (TNM) and pathological grading

		**No**	**%**
**T**	T1	3	12.5%
	T2	13	54.2%
	T3	6	25.0%
	T4	2	8.3%
**N**	N0	20	83.3%
	N1	3	12.5%
	N2	1	4.2%
**M**	M0	23	95.8%
	M1	1	4.2%
**Mitotic count**	1	14	58.3%
	2	10	41.7%
**Tumor necrosis**	No	14	58.3%
	Yes	10	41.7%
**Grading/TNM**	Grade 1	1	4.2%
	Grade 2	16	66.7%
	Grade 3	7	29.2%
**Grading/FNCLCC**	Grade 1	7	29.2%
	Grade 2	11	45.8%
	Grade 3	6	25.0%

Primary surgical treatment was done for 20 patients (83.33%) with R0 margin was achieved in 6 patients (25%), R1 margin in 11 patients (45.8%) and only 3 patients had R2 resected margins (12.5%). Radiotherapy was given as adjuvant treatment in 9/24 patients, as definitive in 2/24 and as palliative in one patient.

Chemotherapy was given in 5 patients as adjuvant chemotherapy and 3 patients received neoadjuvant chemotherapy. The main chemotherapy protocol used was VAC (vincristine, adriamycin, cyclophosphamide) as was given to 4 patients. Two patients received (cisplatin and adriamycin) combination protocol, one received (ifosfamide and adriamycin) combination protocol and one received VAC-IE (vincristine, adriamycin, cyclophosphamide alternating with ifosfamide, etoposide). Eight patients had local recurrence (8/20) and 3 systemic metastasis (3/23). By the end of the study, 17 patients were still alive (70.8%) (Table 3).

**Table 3:** Relapsed disease, site of relapse and palliative treatment

		**No**	**%**
**Relapsed/ Not**	No	9	37.5%
	Relapsed	9	37.5%
	NA	6	25.0%
**Local recurrence**	No	12	50.0%
	Yes	8	33.3%
	NA	4	16.7%
**Systemic metastasis**	No	20	83.3%
	Yes	3	12.5%
	NA	1	4.2%
**Surgery of LR/Palliative amputation**	No	17	70.8%
	Yes	5	20.8%
	NA	2	8.3%
**L.R/palliative radiotherapy**	No	22	91.7%
	Yes	1	4.2%
	NA	1	4.2%
**1st line palliative Cth**	No	15	62.5%
	Ifosfamide/ Adriamycin	2	8.3%
	Liposomal doxorubicin	1	4.2%
	VACA^a^	1	4.2%
	Ifosfamide/ Etoposide	1	4.2%
	Cisplatin/Etoposide	1	4.2%
	NA	3	12.5%
**Alive/death**	Alive	17	70.8%
	Died	7	29.2%

^a^*VAC* Vincristine, adriamycin, cyclophosphamide

Mean time to relapse (TTR) of studied patients was 39.117 months with 95% CI (26.708 to 51.526), mean time to local relapse was 41.76 months with 95% CI (29.561 to 53.962) while mean time to systemic relapse was 56.08 months with 95% CI (46.790 to 65.369) (Table 4).

**Table 4:** Shows estimated time to overall relapse, time to local relapse and time to systemic relapse of studied patients

	Number of events a1*		Number censored b1*		Number of events a2**		Number censored b2**		Number of events a3***		Number censored b3***	
	***N***	**%**	***N***	**%**	***N***	**%**	***N***	**%**	***N***	**%**	***N***	**%**
	9	37.50	15	62.50	8	33.33	16	66.67	3	12.50	21	87.50
**Total number**	24				24				24			
**Mean (mon.)**	39.117				41.761				56.079			
**95% CI for the mean**	26.708 to 51.526				29.561 to 53.962				46.790 to 65.369			
**SE**	6.331				6.225				4.739			

^*^a1 TTR event = 1, b1 TTR event = 0

^**^a2 TTLR event = 1, b2 TTLR event = 0

^***^a3 TTSR event = 1, b3 TTSR event = 0

**Fig. 3:**
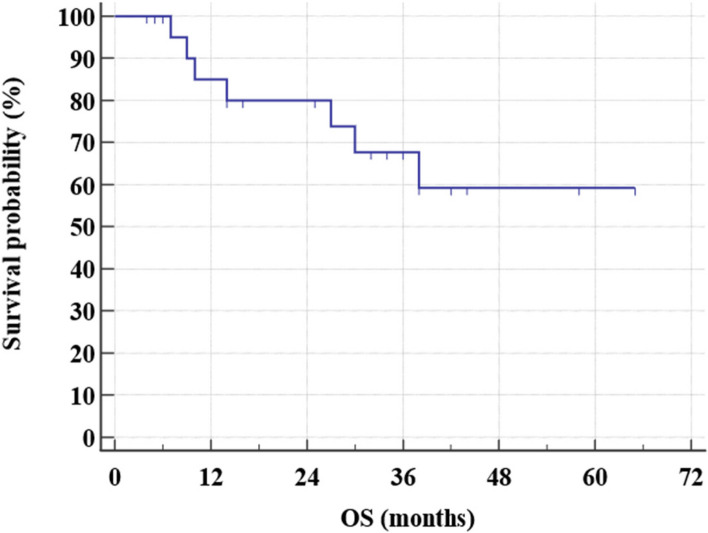
Overall survival curve of studied patients according to Kaplan Mayer analysis

Regarding overall survival (OS) of patients of our study, Mean OS was 47.223 months with 95% CI (36.668 to 57.778) (Table 5, Fig. 3). The tumor grade, necrosis, margin, and adjuvant chemotherapy showed a trend to improve the OS but did not reach statistical significance, which may be related to the small sample size, and the 5-year OS was 65.4%. So, correlative survival analysis between different clinic-pathological factor and survival was difficult.

**Table 5:** Shows estimated overall survival (OS) of studied patients

	Number of events a*		Number censored b*	
	***N***	**%**	***N***	**%**
	7	29.17	17	70.83
**Total number**	24			
**Mean (mon.)**	47.223			
**95% CI for the mean**	36.668 to 57.778			
**SE**	5.385			

^*^a OS event = 1, b OS event = 0

## Discussion

Sarcomas represent a group of very rare diseases, with great diversity, which makes it difficult to run survey studies. This is a retrospective study of clinic-pathological and treatment related factors of patients diagnosed with head and neck sarcomas and the impact on survival. Twenty-four patients are included in the study. A comparative statistically significant results are done with other related studies.

A cross-sectional, descriptive study, with retrospective data of 36 patients with primary sarcomas of the head and neck, seen between 1999 and 2008 in the Head and Neck Surgery Centers in Brazil, mean age of the patients was 39.7 which is younger than our mean patients ‘age 48. Regards gender, 23 were males, and 13 were females while in our study there is female predominance (9 males and 15 females). Like our study, the most common pathological type was rhabdomyosarcoma. By the end of this study, 66.6% of patients were alive which represented a close percentage relative to our study as we had 70.8% of studied patients were still alive [8].

Patients had head and neck soft tissue sarcoma and osteogenic sarcoma treated at the Stomatology Hospital of Nanjing University between 2008 and 2018 were retrospectively analyzed. Sixty-three patients were diagnosed with head or neck sarcoma of which 42.9% had soft tissue sarcoma and 57.1% had osteogenic sarcoma. Of soft tissue sarcoma patients, the most frequently observed histopathologies were fibrosarcoma and malignant fibrous histiocytoma [9]. These results are close to ours as osteosarcoma is the commonest presenting pathological subtype, but soft tissue sarcomas presentation was different as rhabdomyosarcoma was the commonest and less common fibrosarcoma, synovial sarcoma, DFS, and angiosarcoma. Radical surgical resection was performed on 56 patients (88.89%) compared to our results (83.33%). For 33 patients, resection and radiotherapy were used (58.92%) compared to our results (45%). Within the observation period, 17 patients died (26.98%) compared to our results (29.2%).

A retrospective cohort study comprised 63 patients with head or neck soft tissue sarcoma treated at the Helsinki University Hospital between 2005 and 2017 [10]. The average age at diagnosis was 53 years with 38 males and 25 females while in our study mean age was about 48 years and female predominant gender with almost reversed ratio (9 males and 15 females). Like our results, the most frequent histological subtype was rhabdomyosarcoma. Seven patients (11%) had neck LN metastasis, and four (6.3%) had distant metastasis at presentation while in our study 4 patients had neck LN metastasis (16.7%) and only one patient had distant metastasis at presentation (4.2%). The 3-year overall survival (OS) was 68% compared to our results 3-year overall survival (OS) was 74.5%. Overall, (41%) patients died during follow-up compared to our results (29.2%) mostly due to longer duration.

## Conclusion

Head and neck sarcomas are very rare challenging malignancies that need a multi-disciplinary team for proper management. Immunohistochemical and molecular analyses are important for pathological diagnosis. Correlative survival analysis between different clinic-pathological factor and survival was difficult due to small sample size with inaccurate unvaluable results. Surgery remains the mainstay of treatment with high rate of local failure due to anatomical constrain. Data remains limited and choice of treatment should be within the focus of clinical multi-center studies.

## Data Availability

Available on demand.

## Abbreviations

DFS: Dermatofibrosarcoma protuberans
TTLR: Local relapse
OS: Overall survival
TTSR: Time to systemic relapse
TTR: Time to relapse
